# Auditory processing performance in blind people

**DOI:** 10.1590/S1808-86942011000400015

**Published:** 2015-10-19

**Authors:** Ludmilla Vilas Boas, Lílian Muniz, Silvio da Silva Caldas Neto, Mariana de Carvalho Leal Gouveia

**Affiliations:** 1 Doctoral student in cognitive psychology, UFPE. Speech therapist; 2 Doctoral degree in cognitive psychology, UFPE. Adjunct professor at the UFPE; 3 Senior Associate Professor - FMUSP. Adjunct professor, UFPE; 4 Doctoral degree in sciences - otorhinolaryngology. Adjunct professor of otorhinolaryngology at the UFPE. Head of the Otorhinolaryngology Unit of the Agamenon Magalhães Hospital

**Keywords:** blindness, hearing, hearing disorders

## Abstract

**Abstract:**

Hearing has an important role in human development and social adaptation in blind people.

**Objective:**

To evaluate the performance of temporal auditory processing in blind people; to characterize the temporal resolution ability; to characterize the temporal ordinance ability and to compare the performance of the study population in the applied tests.

**Methods:**

Fifteen blind adults participated in this study. A cross-sectional study was undertaken; approval was obtained from the Pernambuco Catholic University Ethics Committee, no. 003/2008.

**Results:**

Temporal auditory processing was excellent - the average composed threshold in the original RGDT version was 4.98 ms; it was 50 ms for all frequencies in the expanded version. PPS and DPS results ranged from 95% to 100%. There were no quantitative differences in the comparison of tests; but oral reports suggested that the original RGDT original version was more difficult.

**Conclusion:**

The study sample performed well in temporal auditory processing; it also performed well in temporal resolution and ordinance abilities.

## INTRODUCTION

By not perceiving visual stimuli, blind individuals develop other abilities, such as improved hearing,^1^ which has an important role in social development and adaptation from the beginning of life. During the sensorimotor period - from birth until 2 year of age - blind children need to experience many auditory and tactile stimuli so that hearing and tactile perception may develop jointly to facilitate locomotion: rolling, dragging, quadrupeding, balancing, supporting, and walking^2^. Auditory sensory information should become integrated with that of other sensory pathways to build future practical intelligence, the notion of objects, spatial organization, and speech acquisition^3,^^4^.

Adequate auditory stimulation is thought to be necessary the social development of blind children and adults; thus, improved care is needed along these lines. There are few published studies on the prevalence of profound visual loss; it is considered as 0.3 to 1.5 per 1,000 children. This paucity of data appears to apply also to hearing loss in Brazil, and to studies that combine these sensory losses^5^.

Thus, given the lack of studies on this topic, the purpose of the present study was to assess temporal processing in blind subjects, and if in fact their dependence on hearing would positively affect their performance in temporal auditory processing tests^1^.

Our goals were as follows: to assess temporal processing performance in blind subjects; to characterize time and frequency temporal resolution abilities by applying the original and extended version of the random gap detection test (RGDT); to characterize temporal ordering ability by applying the duration pattern test; to characterize temporal ordering in blind subjects by applying the frequency pattern test; and to compare the performance of the study population in applied processing tests. We expect this study to contribute by informing blind subjects about the importance of constant stimulation and auditory prevention to improve communication and spatial orientation.

## MATERIAL AND METHODS

This study was carried out in a teaching clinic for speech therapy students at a private university in the city of Recife (PE). The sample consisted of 15 male and female blind adults aged over 18 years, who attend a child education center (Centro de Educação Infantil Professor Gildo Soares da Silva), and who volunteered to participate. The inclusion criteria were: being native Portuguese-speakers; having normal auditory acuity (air conduction thresholds up to 20 dBHL at 500 to 4,000 Hz^6^; type A tympanograms^7^; present stapedial reflexes ipsi- and contralaterally at all frequencies and with gaps detection thresholds below 20 milliseconds (ms) in the subtest 1 of the RGDT as proposed by Keith^8^; normal linguistic development; regularly enrolled in the above mentioned child education center.

Subjects with other disorders or syndromes that affected their understanding of the tests were excluded, as were musically trained individuals, and users of medication that affected the central nervous system. A descriptive cross-sectional study was carried out during two months.

The institutional review board of the Pernambuco Catholic University reviewed our institution's study proposal, which was accepted (protocol no. 003/2008). Before testing, and with a witness present, participants read an introductory letter and read and signed a free informed consent form.

Procedures were scheduled after formally contacting the teaching speech therapy clinic and the child education center. The first step was to take a clinical history based on Pereira & Schochat^9^ and Santos et al.'s^10^ models. Next, otoscopy was carried out (Welch Allyn 29000 otoscope). Subjects were selected after immittance testing (Interacoustics AZ7 immittance tester), which consisted of a tympanometric study and evaluation of ipsilateral stapedial reflexes at 1,000 and 2,000 Hz and contralateral reflexes at 500, 1,000, 2,000, and 4,000 Hz.^7^ The following step was pure tone audiometry at 500 to 4,000 Hz (sloping technique on a 10 dBHL scale^6^), temporal auditory processing, and voice audiometry, for the speech recognition percentage rate and speech recognition threshold (all with an Amplaid model 460 audiometer).

Over-the-ear TDH 39 headphones were used throughout. A Panasonic model S 35 compact disc (CD) player was coupled to the audiometer for specific temporal auditory processing tests. The RGDT, proposed by Keith^8^, was used for temporal processing testing (central auditory evaluation); it consisted of material recorded on a CD at a comfortable intensity as reported by participants. The first step was subtest 1, to assure that the procedure was understood (selection of subjects), followed by subtests 2 and 3 to assess temporal processing (data gathering).

Click pairs (17 ms duration) were presented to measure the shortest time interval or gaps, in milliseconds, at which subjects identified two tones. Subtest 1 was done at 500 Hz only; subtest 2 was done at 500, 1,000, 2,000, and 4,000 Hz, in 0 to 40 ms gaps presented randomly. Subtest 3 (extended version) differed from subtest 2 only in the gap values, in this case from 50 to 300 ms. Gap detection thresholds up to 20 ms in subtest 2, per frequency, are considered normal.

Three of four normal analyzed frequencies were needed so that there was no evidence of temporal processing disorders. The compound RGDT was calculated by taking the mean response value at four tested frequencies^8^.

The frequency pattern test (FPT) comprised 30 presentations consisting of three stimuli at different frequencies at 50 dBSL. The last was the duration pattern test (DPT) comprising 30 presentations consisting also of three stimuli of varying duration at 50 dB SL.

The desired response in both tests was for participants to imitate the stimuli. The minimum expected result in adults is 85% correct answers (FPT)^11^ and 67% correct answers (DPT).^11^ Audiometry and temporal processing tests were done in acoustic booths; the materials for central and peripheral audiologic assessments were calibrated according to ASHA parameters^6^. At the end of the study, a feedback talk was offered to the participants; this presentation consisted of guidelines about the importance and need for continuous auditory stimulation and prevention to improve communication and spatial orientation.

Data analysis consisted of descriptive statistics - using computer tools (Microsoft Office Excel 2003) - to record, organize, and compare the results. These were discussed and presented in charts, and compared with the published speech therapy literature.

## RESULTS

The study sample comprised 15 subjects; three were excluded because their auditory thresholds were above 20 dB at one or more frequencies. The remaining 12 subjects (100%) had results within normal limits - temporal resolution and temporal ordering - as adopted in this study. These results show excellent performance in temporal processing in the study sample - mean compound threshold = 4.98.

The original version of the RGTD showed that gap detection thresholds at each frequency were below 20 ms, as were the compound gap thresholds in the study sample (Frame 1). The smallest means were found at 500 (3.38 ms) and 1,000 (4.58 ms); the highest value was seen at 2,000 Hz (6.16 ms) (Frame 1). The RGDT-E (extended version) showed a shorter gap - 50 ms - per frequency and compound threshold, for all subjects (Frame 2). Performance was within normal limits throughout the sample in the duration pattern test and the frequency pattern test; results ranged from 95 to 100% in most subjects, which was evidence of good performance in temporal ordering of non-verbal sound abilities (Tables 1 and 2).

**Frame 1:** Analysis of random gap detection thresholds per frequency and compound thresholds for each participant, and the group mean - using the RDTG (N=12).

Threshold per Frequency (U+0002Ams)	500 Hz	1,000 Hz	2,000 Hz	4,000 Hz	Compound threshold (U+0002Ams)
1	2	10	10	10	8
2	2	5	10	10	6.75
3	2	5	5	5	4.25
4	2	2	10	5	4.75
5	5	10	5	5	6.25
6	2	2	2	5	2.75
7	2	2	2	5	2.75
8	5	5	10	5	6.25
9	2	2	10	5	4.75
10	2	2	2	5	2.75
11	2	5	5	5	4.25
12	2	5	5	5	4.25
Mean for the group (N=12)	3.38	4.58	6.16	5.83	4.98

* ms - milliseconds

**Frame 2:** Analysis of random gap detection thresholds (extended) per frequency and compound thresholds (extended) for each participant, and the group mean - using the RDTG - E (N=12).

Threshold per Frequency (U+0002Ams)	500 Hz	1,000 Hz	2,000 Hz	4,000 Hz	Compound threshold (U+0002Ams)
1	50	50	50	50	50
2	50	50	50	50	50
3	50	50	50	50	50
4	50	50	50	50	50
5	50	50	50	50	50
6	50	50	50	50	50
7	50	50	50	50	50
8	50	50	50	50	50
9	50	50	50	50	50
10	50	50	50	50	50
11	50	50	50	50	50
12	50	50	50	50	50
Mean for the group (N=12)	50	50	50	50	50

* ms - milliseconds

**Table 1:** Analysis of results of the duration pattern test, per ear, for all participants (N=12).

Results as percentage of correct answers	86.67%	93.33%	96.66%	100%	Total no. of ears
Right ear	0	1	5	6	12
Left ear	1	0	3	8	12

**Table 2:** Analysis of results of the frequency pattern test, per ear, for all participants (N=12).

Results as percentage of correct answers	85%	90%	95%	100%	Total no. of ears
Right ear		1	7	3	12
Left ear	0	1	2	9	12

As no subject had subnormal results in any test, we were unable to characterize degrees of performance (worse or better) for the auditory processing tests that were used. Although results were generally excellent, verbal reports have suggested that the original RGDT was more difficult to carry out.

## DISCUSSION

According to the reference literature, the original version of the RGDT test defines the normal gap detection as being 20 ms or less at all frequencies^8,^^12,^^13,^^14^. This was observed in our study, where values were less than half the normal value for the tested abilities - on average a 4.98 compound threshold - which revealed significant temporal resolution agility in the study sample.

Ballen et al.^15^ evaluated normal hearing children and found that the mean compound threshold was 10.94 ms. Ziliotto & Pereira^16^ applied the RGDT in 236 subjects (normal hearing and with hearing loss) aged from 5 to 53 years, and found that the mean gap detection threshold in the normal auditory processing group was 6.74 ms. These authors suggested that the normal mean gap detection threshold was 7.32 ms or less; thus, any value above this level could be considered as altered.

Even with this reference value, the mean compound threshold in the study samples was lower. Auditory and tactile stimuli are perceived as alternatives for exploring and interacting with the world during the first years of life of visually challenged children; they offer an alternative potential motivation besides vision for children to interact with objects^5^. Our data reinforce the idea that children who become blind before 2 years of age develop their hearing better to compensate the loss of vision, as has been shown in a Canadian study at the Montreal University Research Center in Neuropsychology and Cognition^1^.

Temporal resolution depends on two processes: analysis of temporal patterns in each frequency channel (intrachannel temporal analysis), and comparison of temporal patterns in each activated auditory channel at each moment (interchannel temporal analysis). These channels refer to the filtering characteristics of the peripheral hearing system.

The cochlea behaves as a set of filters (named “auditory filters”) that separate the components of a complex signal into “channels” of different central frequencies. Temporal analysis may be considered as the result of these two main processes^17^. More peripheral frequencies - taking the central nervous system as the reference point - take longer to reach the auditory cortex.

The best performance was at 500 Hz in the original version of the RGDT, which corroborates previous studies^8,^^12,^^13^ showing the influence of auditory pathway tonotopy on temporal resolution. The literature also contains results^18^ with different behaviors, showing that this may not be the standard behavior, although it would be expected.

The extended version of the RGDT is used for detecting silent gap thresholds within sound that are presented to individuals, when these gaps are not measured in the original version^18^. For this reason, the sampled thresholds are the shortest gaps, which corroborates any normalcy encountered in the original version.

FPT and DPT results ranged from 95 to 100% in most of the study sample, once again showing good acoustic information and processing performance in temporal ordering ability. Prando et al. (2010)^19^ applied the frequency pattern test to study teenagers of both sexes with no past or present associated neurological, psychiatric, visual, auditory, and/or linguistic disorders, and no history of alcohol, drug, or benzodiazepine abuse. These researchers found that the mean number of correct answers in imitation mode was 88.88%.

Visual loss does not alter development, but requires other sensory strategies to communicate and to build a mental representation of the world^5^. This may help develop auditory abilities, which explains a good performance in attention and memory, which is the case of the FPT.

Stevens & Weaver^20^ used magnetic resonance imaging to show that functional responses in auditory cortical areas are altered in blind individuals; such intramodal auditory plasticity may indicate that blind subjects have superior auditory perception, and that this advantage may be observed in the behavioral test we applied in the present study. The neural basis of human frequency perception - even in individuals with no sensory changes - is not entirely known; researchers have speculated that the right auditory cortical region is more specific for frequency resolution compared to its left homologous region.

Hyde et al.^21^ demonstrated this concept by studying healthy individuals with functional magnetic resonance imaging. This was not observed in our sample when assessing higher percentages of correct answers to the FPT. Moore et al.^22^ compared the performance of children in frequency discrimination tasks in several listening situations, and emphasized that lack of attention is one of the main causes of poor performance. On the other hand, early on blind people learn to focus their attention on ambient auditory stimuli, which may explain their excellent performance in non-verbal tests in the present study.

Auditory processing comprises the successive processes whereby subjects carry out acoustic and meta-cognitive analyses of sounds^23^. Temporal abilities are also essential for perception of music, rhythm, punctuation, pitch discrimination, and duration of phonemes^24^. In general, blind persons and listeners require good quality temporal processing not only to locate themselves in space but to learn oral language.

Visual loss interferes in social relations in terms of perception of needs and communication, as gazing provides important signals to generate synchronized affective and interactive behavior cycles. Healthcare professionals have a role in helping blind subjects to discover alternative sensory reception pathways. Thus, exploring auditory pathways becomes a relevant alternative.^5^

## CONCLUSION

The study sample performed highly in temporal processing tests irrespective of blindness. Lower values were found when applying the original version of the RGDT for the temporal resolution ability; the reference values at 500 to 4,000 Hz were: 3.38, 4.58, 6.16, and 5.58 ms.

The value at all tested frequencies in the extended RGDT was 50 ms. The temporal ordering ability values ranged from 95 to 100%, taking into account the FPT and the DPT, which were normal.

There were no numerical differences among the procedures, as subjects performed well in all of them; however, verbal reports from participants suggested that the original version of the RGDT was more difficult to carry out.
